# LC3-Mediated Mitophagy After CCCP or *Vibrio splendidus* Exposure in the Pacific Oyster *Crassostrea gigas*

**DOI:** 10.3389/fcell.2022.885478

**Published:** 2022-05-20

**Authors:** Jiejie Sun, Xiaoqian Lv, Jinyuan Leng, Lingling Wang, Linsheng Song

**Affiliations:** ^1^ Liaoning Key Laboratory of Marine Animal Immunology, Dalian Ocean University, Dalian, China; ^2^ Liaoning Key Laboratory of Marine Animal Immunology & Disease Control, Dalian Ocean University, Dalian, China; ^3^ Southern Marine Science and Engineering Guangdong Laboratory, Zhuhai, China; ^4^ Dalian Key Laboratory of Aquatic Animal Disease Control, Dalian Ocean University, Dalian, China

**Keywords:** mitophagy, LC3, CCCP, *Vibrio splendidus*, *Crassostrea gigas*

## Abstract

Mitochondrial selective autophagy, known as mitophagy, surveils the mitochondrial population by eliminating superfluous and/or impaired organelles to mediate cellular survival and viability in response to injury/trauma and infection. In this study, the components of the mitophagy pathway in the Pacific oyster *Crassostrea gigas* were screened from NCBI with reference to the protein sequences of the human mitophagy process. A total of 10 mitophagy process–related genes were identified from *C. gigas*, including NIX, FUNDC1, PHB2, Cardiolipin, P62, VDAC2, MFN2, PARL, MPP, and OPTN. They shared high similarities with their homologs in the human mitophagy pathway and were expressed in various tissues of *C. gigas*. After CCCP exposure, the fluorescence intensity of the mitochondrial probe JC-1 monomers increased significantly in hemocytes, while the fluorescence intensity of JC-1 aggregates decreased significantly. Meanwhile, the fluorescence of lysosomes was found to be co-localized with that of *Cg*LC3 and mitochondria in CCCP-treated hemocytes. Double- and single-membrane-bound vacuoles resembling autophagic structures were observed in the hemocytes after CCCP exposure. The fluorescence intensity of JC-1 monomers and the abundance of *Cg*LC3Ⅱ in hemocytes both increased after *Vibrio splendidus* exposure. At the same time, the green signals of *Cg*LC3 were co-localized with red signals of the mitochondria, and the fluorescence intensity of autophagy increased significantly in hemocytes after *V. splendidus* exposure. The results confirmed the existence of a complete mitophagy pathway in mollusks for the first time, which was helpful for further study on the function of mitochondrial autophagy in mollusks.

## Introduction

Degradation of mitochondria *via* a selective form of autophagy, named mitophagy, is a fundamental mechanism conserved from yeast to humans (Onishi, et al., 2021). There are various proteins and other molecules to modulate mitophagy, and mitophagy is involved in many processes, such as cellular differentiation, inflammation, and apoptosis (Baechler, et al., 2019; Cervantes-Silva, et al., 2021; Onishi, et al., 2021). The mitophagy process starts when the dysfunctional mitochondria are targeted with specific receptors or adapters and engulfed in a double-membrane vacuole named autophagosome. The vesicle fuses with a lysosome to form an autolysosome in which specific enzymes degrade the organelle (Harper, et al., 2018; Poznyak, et al., 2021).

There are two pathways, the ubiquitin-dependent pathway and receptor-dependent pathway, for mitophagy to serve as a critical quality-control mechanism for selective targeting and removal of damaged or dysfunctional mitochondria (Huang and Klionsky, 2021; Nakamura, et al., 2021). The two pathways have been identified in yeast, plants, and mammals (Gan, et al., 2018; Gkikas, et al., 2018; Huang and Klionsky, 2021; Nakamura, et al., 2021; Ren, et al., 2021). The ubiquitin-dependent mitophagy is characterized by PINK1/Parkin-mediated ubiquitination of the mitochondrial proteins, including optineurin (OPTN), sequestosome 1 (SQSTM1, also known as P62), TAX1-binding protein 1 (TAX1BP1), nuclear domain 10 protein 52 (NDP52), voltage-dependent anion channel (VDAC), and mitofusin (MFN) (Huang and Klionsky, 2021; Nakamura, et al., 2021; Ren, et al., 2021). The receptor-dependent mitophagy depends on diverse mitophagy receptors, including BCL2 interacting protein 3 (BNIP3), Nip3-like protein X (NIX), FUN14 domain-containing protein 1 (FUNDC1), cardiolipin, and prohibitin 2 (PHB2) (Huang and Klionsky, 2021; Nakamura, et al., 2021; Ren, et al., 2021). Most of the mitophagy-related proteins contain LC3-interacting region (LIR) motifs, which can directly interact with LC3 to promote the engulfment of defective mitochondria. However, there is still no information about the occurrence, pathways, and molecular components of mitophagy in aquatic invertebrates.

Recently, mitophagy was reported to occur in response to some bacterial infection. In vertebrates, *Listeria monocytogenes* in macrophages could induce mitophagy through the virulence factor listeriolysin O (LLO) (Zhang, et al., 2019). *Lactobacillus infantis* infection also resulted in mitochondrial damage and impaired mitophagy in the ileum and porcine intestinal epithelial J2 cells (Xia, et al., 2020). In invertebrates, the information about mitophagy mainly comes from *Drosophila* and *Caenorhabditis elegans*, and mitophagy usually occurs in muscle cells and neuron cells (Aman, et al., 2021; Cornelissen, et al., 2018; Palikaras, et al., 2019). Meanwhile, there are a few genes identified to participate in the mitophagy process of invertebrates, including MFN2, Parkin, and FUNDC1 (Byrne, et al., 2019; Carroll, et al., 2018; Cummins, et al., 2019; Koehler, et al., 2017). However, there is no report on mitophagy in the hemocytes of aquatic invertebrates (Koiwai, et al., 2021; Picot, et al., 2019).

The Pacific oyster, *C. gigas*, is one of the most important cultured mollusks worldwide. It inhabits the estuarine and intertidal regions and is subjected to extraordinary abundant microbial challenges from the surrounding environment. Mitophagy is a critical mechanism necessary to maintain the mitochondrial quality by removing the damaged, old, and dysfunctional mitochondria. Recently, the released whole-genome sequence of Pacific oysters provides a powerful help to explore mitophagy and its regulation mechanism in mollusks (Zhang, et al., 2012). In the present study, the components in mitophagy were screened with the objectives to confirm the existence of the mitophagy pathway in oysters and understand its activation mechanism in response to CCCP treatment or bacterial stimulation.

## Materials and Methods

### Animals, Immune Challenge, and Sample Collection

Pacific oysters, *C*. *gigas* (shell length 12–16 cm) were collected from a local farm in Dalian, Liaoning, China, and cultured in aerated seawater (SW) at 15 ± 2°C for 7 days. Different tissues (hemolymph, adductor muscle, gills, mantle, gonad, labial palps, and hepatopancreas) were collected from nine untreated oysters, and every three individuals were pooled together as one sample. There were three replicates for each tissue. The collected samples were added with TRIzol reagent (Invitrogen) for RNA extraction to examine the relative mRNA expressions of mitophagy-related genes. Hemolymph was collected from the posterior adductor muscle sinus of each oyster by using 1 ml of acid citrate-dextrose anticoagulant agent (20.8 g L^−1^ glucose, 3.4 g L^−1^ EDTA·2Na, 22.5 g L^−1^ NaCl, and 8.0 g L^−1^ disodium citrate) at a ratio of 1:1 and then centrifuged at 800 × *g* at 4°C for 4 min to harvest the hemocytes. The collected hemocytes were treated with 20 µM CCCP (Abcam) at 18°C with a 20 µM DMSO group as control. The samples were then analyzed by using Western blot or immunocytochemical assay.

The hemocytes were also collected after the oysters received an injection of 100 µl *Vibrio splendidus* at 2 × 10^8^ CFU ml^−1^ dissolved in SW. The SW was used as the control. Nine oysters were randomly sampled from each group at 0, 3, 6, 12, 24, and 48 h after *V. splendidus* stimulation. The hemocytes from three oysters were pooled together as one sample, and there were three samples for each time point. The collected samples were then analyzed using Western blot.

### Characterization, Sequence Alignment, and Phylogenetic Relationship Analysis of Mitophagy-Related Genes

Searching of mitophagy-related genes in *C. gigas* transcriptome data was conducted by using the tBLAST program acquired from the NCBI database (https://www.ncbi.nlm.nih.gov/) with known proteins (from mammals, yeast, insects, etc.) as queries. The primers for these genes (Supplementary Table S3) were designed in accordance with the sequence information acquired from the NCBI database (https://www.ncbi.nlm.nih.gov/). The obtained PCR products were inserted into a pMD 19-T vector (TaKaRa) and sequenced by Sangon Biotech (Sangong, China).

A translation tool (http://web.expasy.org/translate/) was used to predict the amino acid sequence of mitophagy-related genes. The simple modular architecture research tool (SMART) (http://smart.embl-heidelberg.de/) was used to predict the conserved domain. ClustalW multiple alignment program (http://www.ebi.ac.uk/clustalw/) and DNAMAN were used to create the multiple sequence alignment. The neighbor-joining (NJ) phylogenetic tree was constructed using the MEGA6.0 package.

### Quantitative Real-Time PCR

The mRNA expressions of mitophagy-related genes in the different tissues were examined by quantitative reverse transcription PCR (qRT-PCR) with the elongation factor (*Cg*EF; NP_001292242.2) fragment amplified with primers *Cg*EF-RT-F and *Cg*EF-RT-R (Supplementary Table S3) as an internal reference. The reaction was carried out in a total volume of 10 μl, including 5 µl of SYBR Green Master Mix (Applied Biosystems), 0.2 µl of each primer, 0.2 µl of ROX, 2 µl of the 20 times-diluted cDNA, and 2.4 µl of DEPC-treated water. qRT-PCR was programmed at 95°C for 10 min, followed by 40 cycles at 95°C for 10 s and 60°C for 45 s. The relative mRNA expression levels were calculated by the comparative Ct method (2^−∆∆Ct^ method) (Livak and Schmittgen, 2001). Significant differences were accepted at *p* < 0.05.

### Western Blot

The hemocyte proteins were extracted from oysters after CCCP treatment or *V. splendidus* stimulation, separated by 15% SDS-polyacrylamide gel electrophoresis, and then transferred onto the nitrocellulose membrane by using a mini transfer tank for electrophoresis. The membranes were blocked with 5% nonfat milk in TBST (10 mM Tris-HCl, pH 7.5, 150 mM NaCl, and 0.2% Tween 20) for 10 h at 4°C and then incubated with 1/1,000 diluted anti-LC3B antibody (ab51520, Abcam) or β-tubulin (Beyotime, China) in TBST, with 5% nonfat milk at 4°C for 10 h. HRP-conjugated Rabbit anti-mouse IgG (Shangon Biotech) diluted at 1/10,000 with TBS (10 mM Tris-HCl, pH 7.5, and 150 mM NaCl) was incubated with the membranes at room temperature for 2 h after the membranes were washed to remove the free nonspecifically bound antiserum. The membranes were finally immersed in the reaction system (1:1 mixing BeyoECL Moon A and B) in the dark for 1 min and imaged with Amersham Imager 600 (GE Healthcare, United States). The relative expression level of *Cg*LC3Ⅱ protein was analyzed by ImageJ with β-tubulin protein as the control (Sun, et al., 2020).

### Mitochondrial Membrane Potential Assay

The hemocytes were extracted from the oysters at 3 h after CCCP treatment or at 24 h after *V. splendidus* stimulation. The hemocytes from the dimethyl sulfoxide (DMSO) or SW group were used as control, respectively. The mitochondrial membrane potential of hemocytes was measured using a mitochondrial membrane potential assay kit with JC-1 (Beyotime, China). The treated hemocytes were deposited on clean slides in the wet chamber and mounted on buffered glycerin (50%) for observation using a fluorescence microscope (ZEISS, Germany). ImageJ was used to calculate the fluorescence signals of JC-1 monomers and JC-1 aggregates.

### Immunocytochemistry

The hemocytes were collected and resuspended in an L15 medium. The suspension was deposited on clean slides (a drop on each) in the wet chamber. After the monolayer was sedimented on the side, the hemocytes were fixed with 4% paraformaldehyde (PFA) for 10 min. After being washed with L15 medium for 5 min, the hemocytes were blocked by incubating in 3% BSA at 37°C for 30 min. The supernatant was then removed, and the dishes were incubated with 500 µl of anti-LC3 (diluted 1:200 in 3% BSA) at 4°C for 2 h. After washing three times with L15 medium, the hemocytes were incubated with Alexa Fluor 488-labeled goat anti-Rabbit secondary antibody (diluted 1:200 in 3% BSA) at 37°C for 1 h. After washing another three times with an L15 medium, the hemocytes were incubated with 2-(4-Amidinophenyl)-6-indolecarbamidine dihydrochloride (DAPI) (Solarbio Life Sciences, China, diluted 1:1,000 in 3% BSA) to stain the nucleus. The slides were mounted on buffered glycerin (50%) and observed using a fluorescence microscope (ZEISS, Germany) after the final three times of washing with the L15 medium. ImageJ was used to calculate the fluorescence signals of *Cg*LC3 in the hemocytes.

### Colocalization of Mitochondria With Lysosomes

An immunocytochemical assay was performed to observe the colocalization of mitochondria with lysosomes in hemocytes 3 h after the treatment with 20 μM CCCP. DMSO was used as the control. A measure of 1 ml of hemocyte resuspension (about 1 × 10^6^ cells in L15 medium) was incubated with MitoTracker Green (Beyotime) and LysoTracker Red (Beyotime) dyes (diluted 1:15,000 in L15 medium) at 37°C in the dark for 30 min, respectively. The hemocytes were washed thrice with L15 medium and fixed with 1 ml of a mixture containing an L15 medium and 4% paraformaldehyde (1:1 in volume) for 10 min. After being washed thrice with L15 medium, the hemocyte resuspension was deposited on poly-lysine microscope slides for adhesion at room temperature for 30 min. Fluorescence was observed under an inverted fluorescence microscope (ZEISS, Germany).

### The Colocalization of Mitochondria and *Cg*LC3, *Cg*LC3, and Lysosome

A measure of 1 mL of the hemocyte resuspension (about 1 × 10^6^ cells in L15 medium) was incubated with LysoTracker Red or mitochondria at 37°C in the dark for 30 min. After being washed thrice with L15 medium, the hemocytes were fixed with 1 ml of a mixture containing an L15 medium and 4% paraformaldehyde (1:1 in volume) for 15 min. The hemocytes were resuspended in an L15 medium and deposited on the poly-L-lysine–coated slides. After 1 h, the slides were incubated successively with 500 µl of anti-LC3B antibody (ab51520, Abcam) (diluted 1:200 in 3% BSA) at 4°C for 2 h and Alexa Fluor 488-labeled goat anti-Rabbit as a secondary antibody (diluted 1:200 in 3% BSA) at 37°C for 1 h. The slides were washed with L15 medium three times and mounted on buffered glycerin (50%) for observation under a fluorescence microscope (ZEISS, Germany).

### Autophagy

The autophagy assay kit (Abcam) was used to analyze the autophagy levels of hemocytes at 24 h after the oysters received an injection of *V. splendidus*. SW was used as the negative control and untreated oysters as the blank control. The hemocytes about 1 × 10^6^ cells mL^−1^ were resuspended in 250 μl 1 × assay buffer and then incubated with 250 μl of diluted CYTO-ID^®^ Green stain solution at room temperature in the dark for 30 min. The treated hemocytes were then washed with 1 × assay buffer three times. The flow cytometry assay was conducted using Amnis ImageStream MkII to count the fluorescence signals of autophagy through IDEAS analysis software. A total of 5,000 hemocytes were analyzed for each sample.

### Transmission Electron Microscopy

The oyster hemolymph was collected from the sinus of five oysters 3 h after CCCP treatment and centrifuged at 1,500 rpm, 4°C for 4 min. The hemocytes were resuspended in an L15 medium. Then, 1 ml of hemocyte suspension (1 × 10^6^ cells) was centrifuged at 1,500 rpm, 4°C for 8 min. The samples were fixed in 3% glutaraldehyde solution (Sigma-Aldrich, G5882) at 4°C for 24 h and then analyzed by JEM-1400 TEM.

### Statistical Analysis

All the data were represented as mean ± standard deviation, and they were analyzed by one-way ANOVA and multiple comparisons using SPSS 22.0. The statistically significant differences were designated at *p* < 0.05 and were extremely significant at *p* < 0.01 (N = 3).

## Results

### Mitophagy in Hemocytes Observed by Transmission Electron Microscopy (TEM) After CCCP-Treatment

**FIGURE 1:**
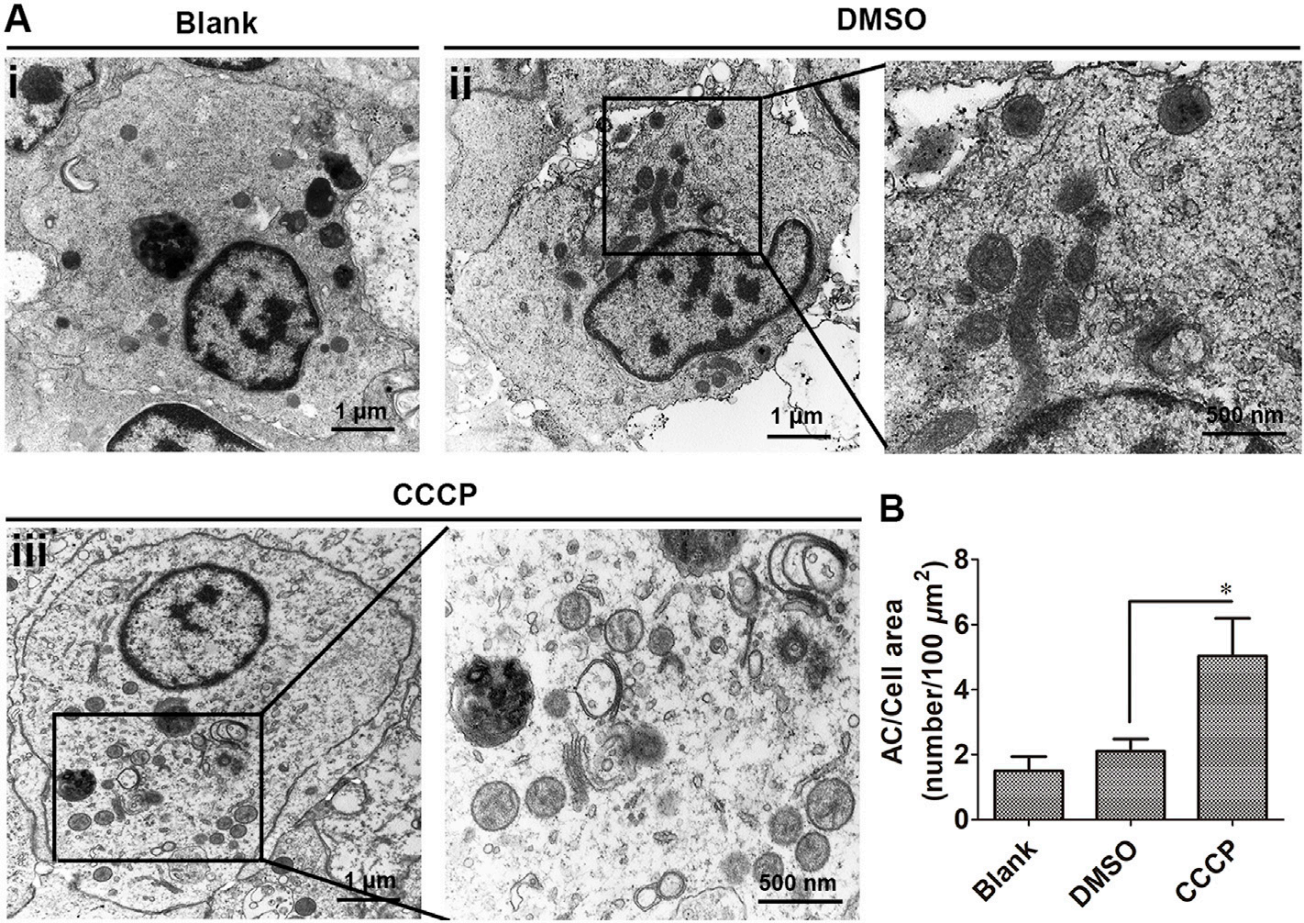
Autophagic ultrastructures in CCCP-treated hemocytes by transmission electron microscopy. **(A)** Autophagic ultrastructures of hemocytes in CCCP-treated hemocytes. AC: autophagosomes. **(B)** Number of autophagosomes in CCCP-treated hemocytes. *: *p* < 0.05 (*t*-test).

The mitophagy inducer CCCP was used to induce mitophagy in oyster hemocytes, and mitophagy was observed by TEM. The organelles were observed to be intact without swelling and deformation in the DMSO group under an electron microscope, and there were few vacuoles present in hemocytes. In the CCCP treatment group, the accumulation of vacuolar structures and autophagosomes was observed in the cytoplasm of hemocytes, which was 2.36-fold (*p* < 0.05) of that in the DMSO group (Figures 1A,B).

### Identification and Sequence Analysis of Ten Mitophagy Process–Related Genes

**FIGURE 2:**
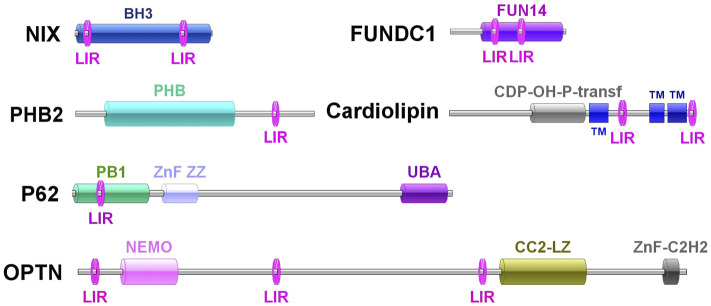
Molecule features of *Cg*NIX, *Cg*FUNDC1, *Cg*PHB2, *Cg*Cardiolipin, *Cg*P62, and *Cg*OPTN and their LIR motifs.

A total of 10 mitophagy process–related genes were identified from *C. gigas*, including *Cg*NIX, *Cg*FUNDC1, *Cg*PHB2, *Cg*Cardiolipin, *Cg*P62, *Cg*OPTN, *Cg*VDAC2, *Cg*MFN2, *Cg*PARL, and *Cg*MPP. They all contained the conserved domains similar to their homologs in mammals and yeast, and their detailed sequence features are shown in Supplementary Table S1. Multiple sequence alignments revealed that the amino acid sequences of PHB2s, cardiolipins, P62s, OPTNs, VDAC2s, MFNs, and MPPs were relatively conserved between all the tested species (Supplementary Figures S1A–S7C). The 10 mitophagy process–related proteins were clustered with their corresponding molecules from *C. virginica*, and all fell in the invertebrate branch in the phylogenetic tree (Supplementary Figures S1B–S7D). *Cg*OPTN contained three LC3-interacting regions (LIRs). *Cg*NIX, *Cg*FUNDC1, and *Cg*Cardiolipin contained two LIRs. One LIR was identified in *Cg*PHB2 and *Cg*P62 (Figure 2).

### Tissue Distribution of the Ten Mitophagy Process–Related Genes

**FIGURE 3:**
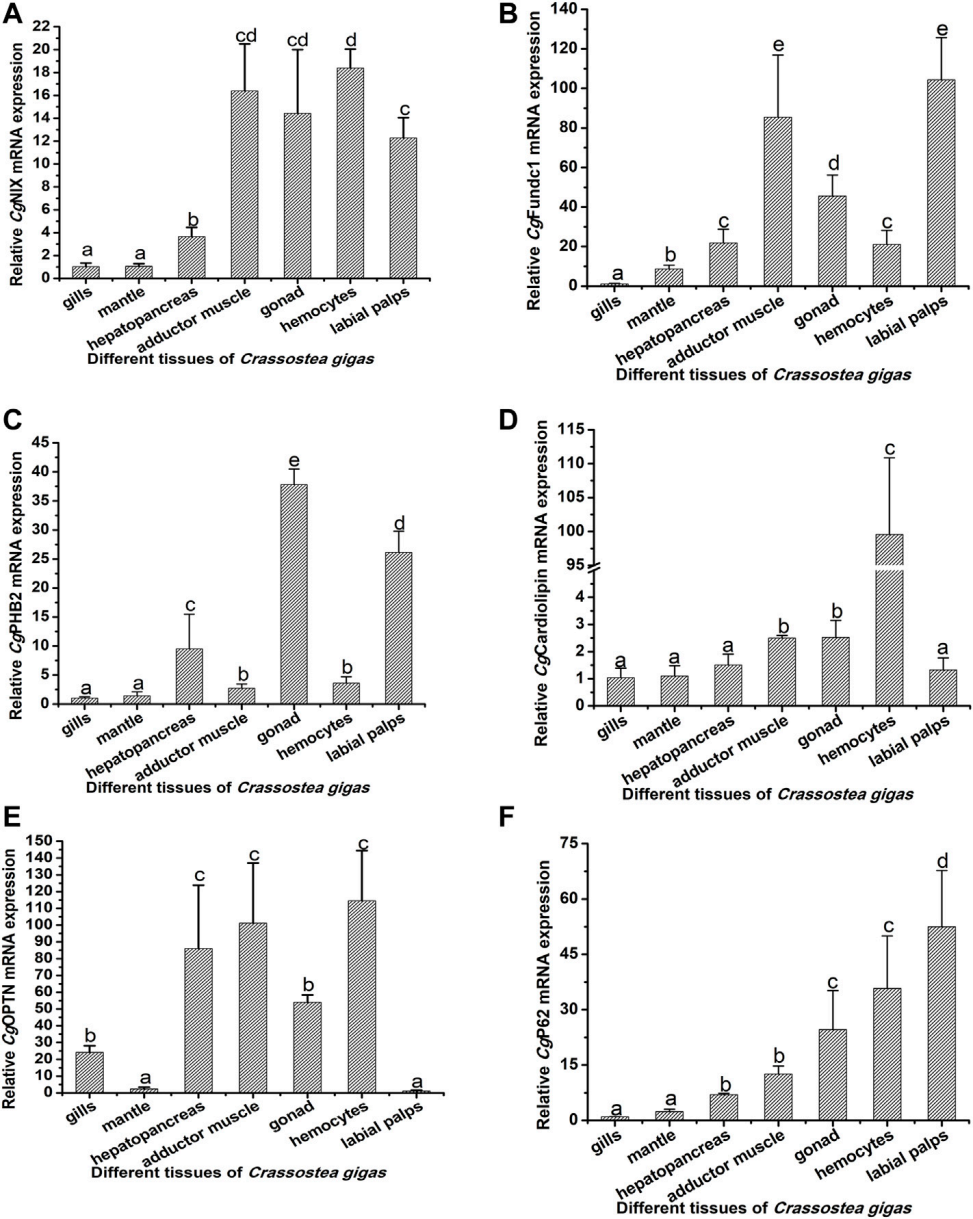
Tissue distribution of *Cg*NIX, *Cg*FUNDC1, *Cg*PHB2, *Cg*Cardiolipin, *Cg*P62, and *Cg*OPTN. The *Cg*EF gene was used as an internal control to calibrate the cDNA template for all the samples. The relative mRNA expression levels of *Cg*NIX **(A)**, *Cg*FUNDC1 **(B)**, *Cg*PHB2 **(C)**, *Cg*Cardiolipin **(D)**, *Cg*P62 **(E)**, and *Cg*OPTN **(F)** in different tissues (the gills, mantle, hepatopancreas, adductor muscle, gonad, hemocytes, and labial palps) were normalized to that of gills. EF was used as the internal control. Vertical bars show as mean ± S.D. (N = 3). The groups that do not share the same letter are significantly different from each other (*p* < 0.05, one-way ANOVA), while the same letters indicate no significant differences among groups (*p* > 0.05).

**FIGURE 4:**
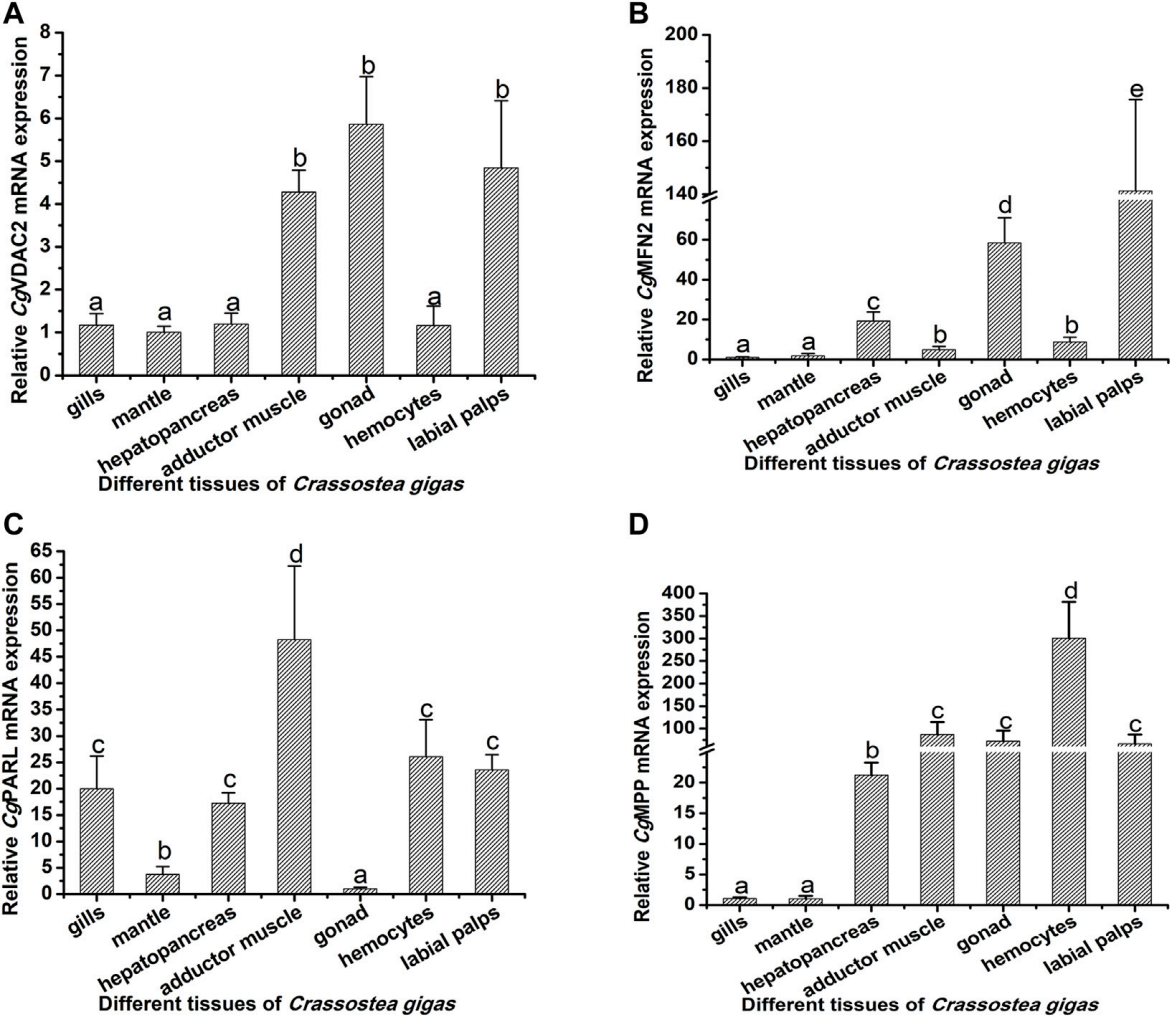
Tissue distribution of *Cg*VDAC2, *Cg*MFN2, *Cg*PARL, and *Cg*MPP. The relative mRNA expression levels of *Cg*VDAC2 **(A)**, *Cg*MFN2 **(B)**, *Cg*PARL **(C)**, and *Cg*MPP **(D)** in different tissues were normalized to that of gills. EF was used as the internal control. The vertical bars are shown as mean ± S.D. (N = 3). Different letters indicate significant differences between groups (*p* < 0.05, one-way ANOVA).

The mRNA transcripts of the 10 mitophagy process–related genes were found to be distributed in all the tested tissues, including the gills, mantle, hepatopancreas, adductor muscle, gonad, hemolymph, and labial palps, with some differences in the expression levels (Figures 3, 4; Supplementary Table S2). The 10 genes had relatively lower expression levels in gills and mantle, and their expression levels were relatively higher in adductor muscle, gonad, and hemocytes (Figures 3, 4; Supplementary Table S2). The detailed information about their mRNA expression levels is listed in Supplementary Table S2.

### Mitochondrial Membrane Potential Change and *Cg*LC3 Activation in Hemocytes in the CCCP Treatment Group

**FIGURE 5:**
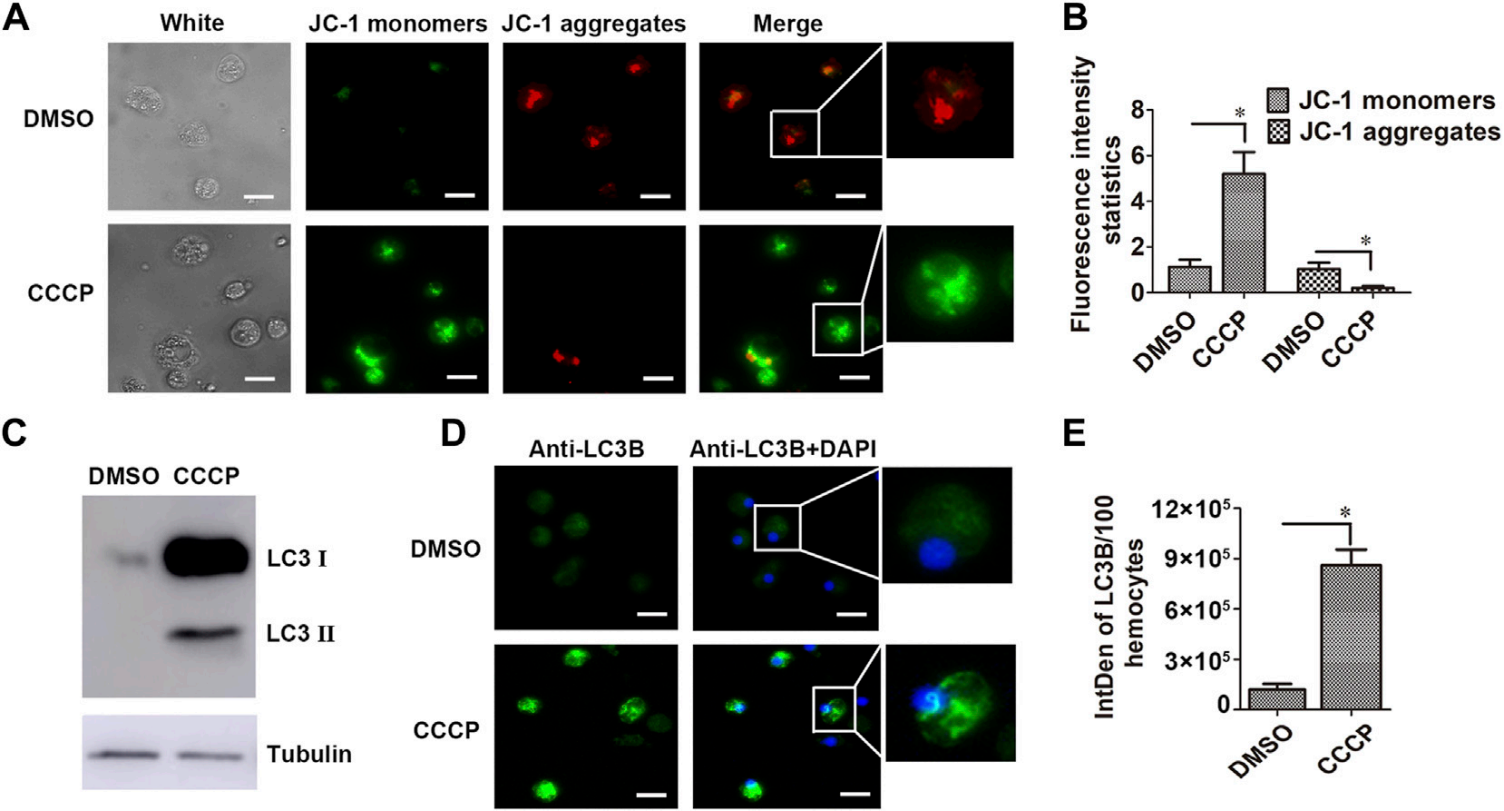
Mitochondrial membrane potential and *Cg*LC3 activation in CCCP-treated hemocytes. **(A)** Mitochondrial membrane potential in CCCP-treated hemocytes. DMSO was used as the control. **(B)** Statistical analysis of the JC-1 monomer and aggregate green signals for A, respectively. **(C)** Cleavage of *Cg*LC3B in CCCP-treated hemocytes. **(D)** Speckled dot accumulation of *Cg*LC3B in CCCP-treated hemocytes. Bar: 5 μm. **(E)** was the statistical analysis of **(D)**. Fluorescence signals were captured under the same exposure parameters. The vertical bars are shown as mean ± S.D. (N = 3). *: *p* < 0.05 (*t*-test).

The mitochondrial membrane potential of hemocytes in the CCCP-treated oysters was determined by using the mitochondrial membrane potential assay kit with the mitochondrial probe 5,5′,6,6′-tetrachloro-1,1′,3,3′-tetraethylbenzimidazolyl-carbocyanine iodide (JC-1). The positive signal of JC-1 monomers in the cell cytoplasm was observed in green, while JC-1 aggregates were stained in red. After CCCP treatment, the fluorescence intensity of JC-1 monomers (green signals) in hemocytes increased significantly, which was 4.60-fold of that in the DMSO group. The value of JC-1 aggregates (red signals) in hemocytes from the CCCP treatment group decreased significantly, which was 0.20-fold of that in the DMSO group (Figures 5A,B). Western blot assay with the *Cg*LC3 antibody revealed that there was a *Cg*LC3B-II band in CCCP-treated hemocytes, while no bands were detected in the DMSO treatment group (Figure 5C). The immunocytochemical assay with the anti-LC3 antibody was used to analyze the subcellular distribution of *Cg*LC3 in the hemocytes in the CCCP treatment group. The positive signals of *Cg*LC3B were observed in green, which were accumulated in hemocytes in the form of speckled dots in the CCCP treatment group, compared with that in the DMSO treatment group (Figures 5D,E).

### Co-localization of Mitochondria With *Cg*LC3 and *Cg*LC3 With Lysosomes

**FIGURE 6:**
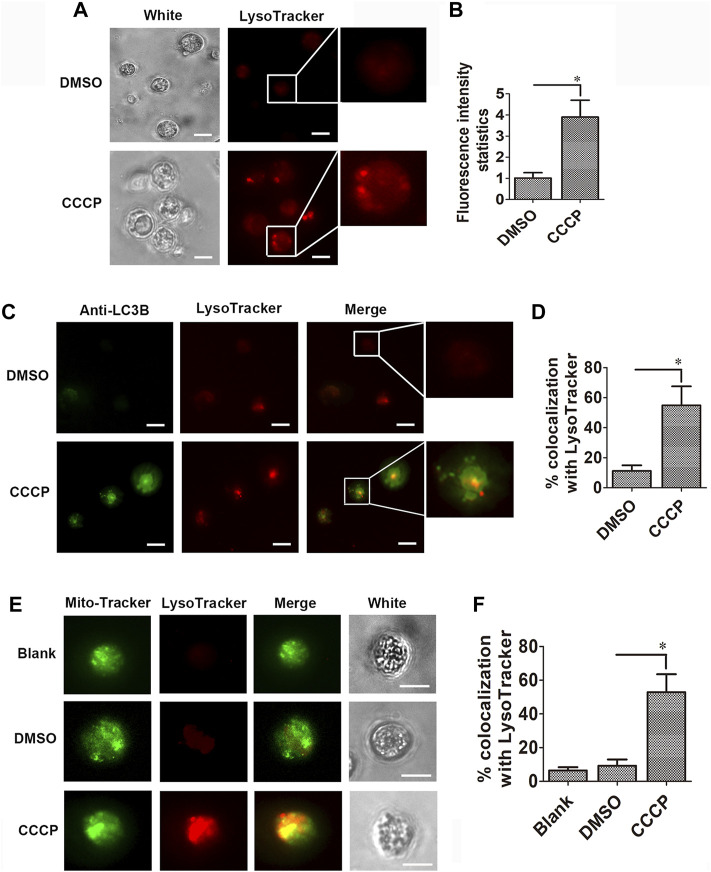
Co-localization of *Cg*LC3 and mitochondria with lysosomes. **(A)** Red signals of lysosomes in CCCP-treated hemocytes. **(B)** Statistical analysis of lysosomes in CCCP-treated hemocytes. **(C)** Co-localization of LC3B with lysosomes in CCCP-treated hemocytes. **(D)** Quantity of LC3B co-localized with lysosomes in CCCP-treated hemocytes. **(E)** Co-localization of mitochondria with lysosomes in CCCP-treated hemocytes. **(F)** Quantity of mitochondria co-localized with lysosomes in CCCP-treated hemocytes. The fluorescence signals were captured under the same exposure parameters. The vertical bars are shown as mean ± S.D. (N = 3). *: *p* < 0.05 (*t*-test).

The content of lysosomes was analyzed with LysoTracker Red in the hemocytes in the CCCP treatment group. The fluorescence intensity of LysoTracker Red–labeled lysosomes (red signals) in the hemocytes of the CCCP treatment group increased significantly, which was 3.91-fold of that in the DMSO group (Figures 6A,B). *Cg*LC3 was marked by the *Cg*LC3 antibody, and Alexa Fluor 488 conjugated antibody was observed in green. In the CCCP treatment group, the statistic value of *Cg*LC3B (green signals) co-localized with the LysoTracker Red–labeled lysosomes (red signals) in hemocytes increased significantly (4.88-fold of that in the DMSO group) (Figures 6C,D). The mitochondria were stained by MitoTracker Green in green signals. The statistic value of mitochondria (green signals) co-localized with the LysoTracker Red–labeled lysosomes (red signals) in the hemocytes of the CCCP treatment group increased significantly, which was 5.73-fold of that in the DMSO group (Figures 6E,F).

### Mitochondrial Membrane Potential Change, *Cg*LC3 Activation, the Co-Localization of *Cg*LC3 With Mitochondria, and the Autophagy Level After *V. splendidus* Stimulation

**FIGURE 7:**
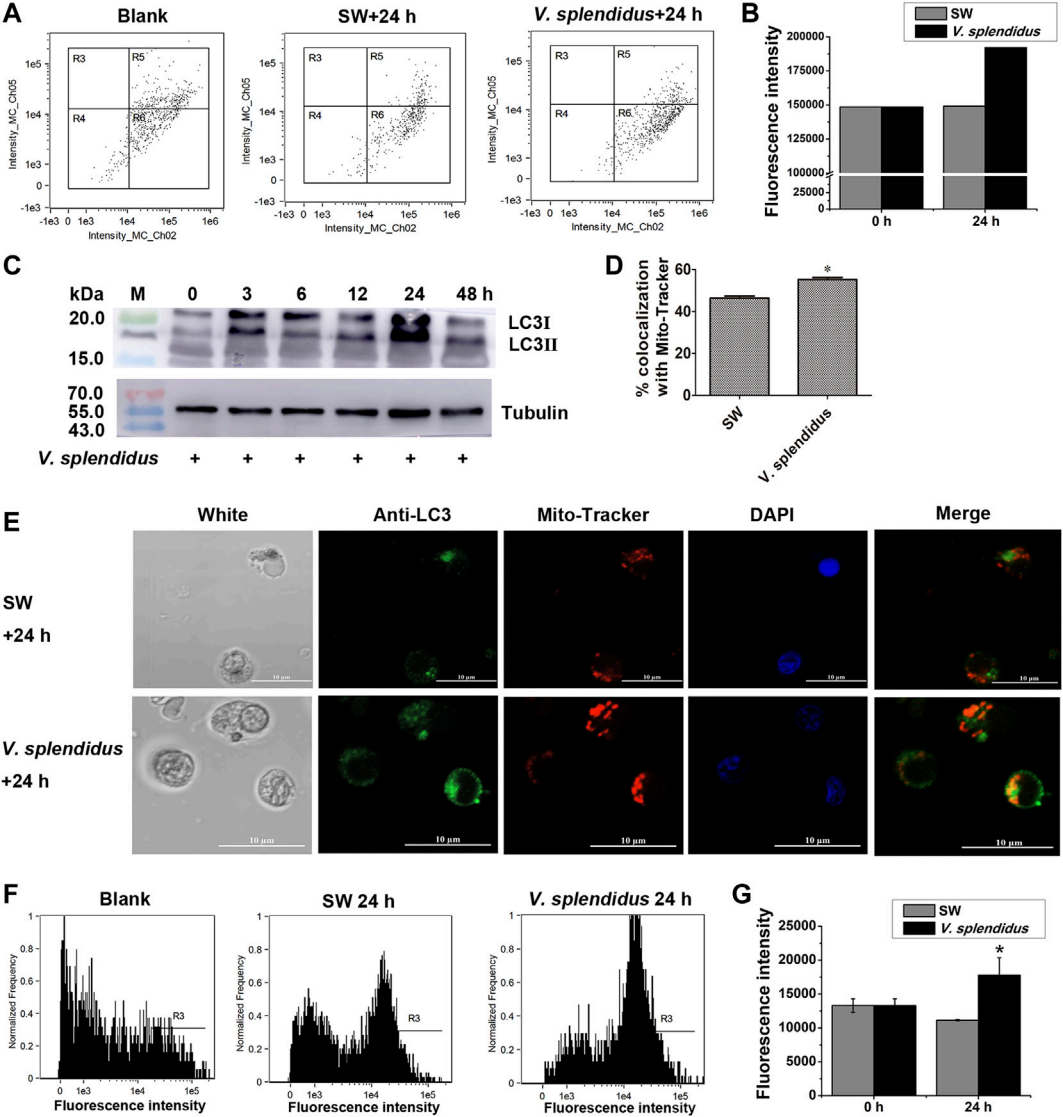
Mitochondrial membrane potential, autophagy, *Cg*LC3 activation, and co-localization of *Cg*LC3 with mitochondria after *V. splendidus* stimulation. **(A)** Mitochondrial membrane potential in hemocytes at 24 h after *V. splendidus* stimulation analyzed by flow cytometry. SW was used as the control. R6: Only green fluorescence of JC-1 monomers (a marker of mitochondrial autophagy). **(B)** Statistical analysis of JC-1 monomer green signals for **(A). (C)** Cleavage of *Cg*LC3B in hemocytes at 0, 3, 6, 12, 24, and 48 h after *V. splendidus* stimulation. Tubulin was used as an internal control. **(D)** Quantity of *Cg*LC3 co-localized with mitochondria in hemocytes after *V. splendidus* stimulation. **(E)** Co-localization of *Cg*LC3 with mitochondria in hemocytes at 24 h after *V. splendidus* stimulation. The fluorescence signals were captured under the same exposure parameters. **(F)** Autophagy in hemocytes at 24 h after *V. splendidus* stimulation. The hemocytes were labeled by CYTO-ID^®^ Green reagent and analyzed by flow cytometry. R3: the cells with green signals. **(G)** Statistical analysis of autophagy green signals for **(F)**. The vertical bars are shown as mean ± S.D. (N = 3). *: *p* < 0.05 (*t*-test).

The fluorescence intensity of JC-1 monomers (green signals) in hemocytes increased after the oysters were stimulated by *V. splendidus*, which was 1.29-fold of that in the SW group (Figures 7A,B). The band intensity of *Cg*LC3B-II in hemocytes increased at 3 and 12 h and peaked at 24 h after *V. splendidus* stimulation (Figure 7C). *Cg*LC3 was marked by the *Cg*LC3 antibody and Alexa Fluor 488 conjugated antibody in green signals, and mitochondria were stained by using MitoTracker Red in red signals. The co-localization of *Cg*LC3 (green signals) with mitochondria (red signals) was enhanced at 24 h after *V. splendidus* stimulation, compared with that of the SW group (Figures 7D,E). The autophagy assay kit was used to analyze the autophagy levels of the hemocytes at 24 h after *V. splendidus* stimulation. The fluorescence intensity of autophagy in hemocytes increased after the oysters were stimulated by *V. splendidus*, which was 1.60-fold of that in the SW group (Figures 7F,G).

## Discussion

The mitochondrion is an organelle essential for multiple biological processes, including energy production, metabolite biosynthesis, cell death, and immunological responses (Cervantes-Silva, et al., 2021; Lou, et al., 2020; Xu, et al., 2021). Mitophagy surveils the mitochondrial population by eliminating superfluous and/or impaired mitochondria and mediating the cellular survival and viability in response to infection (Deretic, 2021; Gkikas, et al., 2018). It is a mechanism widely conserved in the eukaryotes and well described in the model organisms such as yeast and humans (Gkikas, et al., 2018). Although the phenomenon of mitophagy has been reported in *Drosophila* and *C. elegans* (Cornelissen, et al., 2018; Palikaras, et al., 2019), the information about the components and the molecular machinery of the mitophagy pathway in invertebrates remains scarce.

It has been well documented that mitophagy plays an important role in the response to various stress factors (Gkikas, et al., 2018). As mollusks are generally exposed to numerous biotic (pathogens) and abiotic (tide, pollution) stressors (Deretic, 2021), it is very important to elucidate the mitophagy pathway in mollusks. CCCP is an oxidative phosphorylation uncoupler, which can increase the permeability of the mitochondrial membrane to protons and thereby destroy the mitochondrial membrane potential (Jin, et al., 2021). In the present study, the accumulation of vacuolar structures and autophagosomes was observed in the cytoplasm of hemocytes in the CCCP treatment group, indicating that mitophagy occurred in oyster hemocytes. With the release of the genome, the mitophagy pathway has been proposed by data mining in the Pacific oyster *C. gigas* (Zhang, et al., 2012). In the present study, the full-length cDNA sequences of mitophagy process-related genes in *C. gigas* were cloned by PCR. The 10 genes all contained the conserved corresponding domains similar to their homologs in mammals and yeast, and they all fell in the invertebrate branch in the phylogenetic trees. A hallmark of most LC3-interacting proteins is the presence of an LIR motif, which is required for their interaction with LC3 (Onishi, et al., 2021). The requirement for LIR motifs to convey the ability of autophagy-related proteins to interact with LC3 is conserved across the eukaryotes (Chu, 2019; Jacomin, et al., 2020). In this study, *Cg*NIX, *Cg*FUNDC1, *Cg*PHB2, *Cg*Cardiolipin, *Cg*P62, and *Cg*OPTN were identified with one or more LIR motifs, indicating their potential interaction with *Cg*LC3 in oysters. The results suggested the existence of the mitophagy pathway in oysters.

The molecules involved in the mitophagy pathway are reported to be widely distributed in various tissues. In humans, the molecules in the mitophagy pathway had been reported to be distributed in the brain, kidney, breast, and pancreas (Li, et al., 2021), while in the lower vertebrates and invertebrates, there were still no reports about the distribution of molecules in the mitophagy pathway. In the present study, the 10 mitophagy process-related genes were found to be constitutively expressed in the gills, mantle, hepatopancreas, adductor muscle, gonad, hemocytes, and labial palps. Similarly, autophagy-related genes, such as ATG12, ATG9, SQSTM1, Beclin1, and LC3, from *C. gigas* were demonstrated to be constitutively expressed in different tissues (Picot, et al., 2020). The results indicated that mitophagy was an evolutionarily conserved process, and it could occur in different tissues of oysters, indicating the important roles of mitophagy in mollusks.

Mitophagy is a form of macroautophagy (Hansen, et al., 2018), which can be induced by CCCP (Liu and Okamoto, 2021). The exposure of cells to the mitochondrial uncoupler CCCP causes an increase in membrane proton conductance and consequently a loss of mitochondrial membrane potential (Kasianowicz, et al., 1984; Seabright, et al., 2020). However, the information about the induction of CCCP on the mitochondrial membrane potential is still unknown in invertebrates. In the present study, CCCP (20 μM) was found to induce the cleavage of LC3Ⅰ into LC3Ⅱ and also promoted the accumulation of LC3 speckled dots in oyster hemocytes. Apart from this, the value of JC-1 monomers in the hemocytes of the CCCP treatment group increased significantly, while the value of JC-1 aggregates decreased significantly, which indicated that CCCP was able to induce a loss of mitochondrial membrane potential. These results suggested that CCCP (20 μM) was able to induce mitophagy in the oyster hemocytes. In mammals, CCCP is normally used as a mitochondrial autophagy inducer to elicit PINK1-induced mitophagy (Shin and Chung, 2020; Soutar, et al., 2019). In this study, CCCP (20 μM) was able to increase the lysosomes and induce the co-localization of LC3 with lysosomes, as well as the co-localization of mitochondria with lysosomes in hemocytes. The results indicated that CCCP could be used as a mitochondrial autophagy inducer to induce mitophagy, and an index was established to test the occurrence of mitophagy in mollusks.

Mitophagy can be triggered by a pathogen infection (Oh, et al., 2021). For example, *M. tuberculosis* and *M. bovis* were reported to cause similar metabolic rewiring of cells coupled to the activation of mitophagy in humans (Mahla, et al., 2021). Bovine papillomavirus could induce Parkin-mediated mitophagy in urothelial cells (De Falco, et al., 2020). Recently, it was reported that severe acute respiratory syndrome coronavirus 2 (SARS-CoV-2) could also promote the accumulation of LC3 in mitochondria and induce mitophagy (Li, et al., 2022). However, in invertebrates, there were still no reports about the mitophagy induced by pathogens. In the present study, *V. splendidus* was found to induce the JC-1 monomer, the cleavage of *Cg*LC3 (Picot, et al., 2020; Picot, et al., 2019), the co-localization of LC3 with mitochondria, and autophagy in oyster hemocytes. The results demonstrated that pathogen infection was able to induce hemocyte mitophagy in mollusks. In mammals, emerging lines of evidence highlight a crucial role of mitophagy in the regulation of the immune system (Lazarou, 2015). Mitophagy was conserved and functional in oysters, and it was an important defense mechanism against the pathogen *V. splendidus*, associated with mortality outbreaks that affected oyster aquaculture (Moreau, et al., 2015).

## Conclusion

**FIGURE 8:**
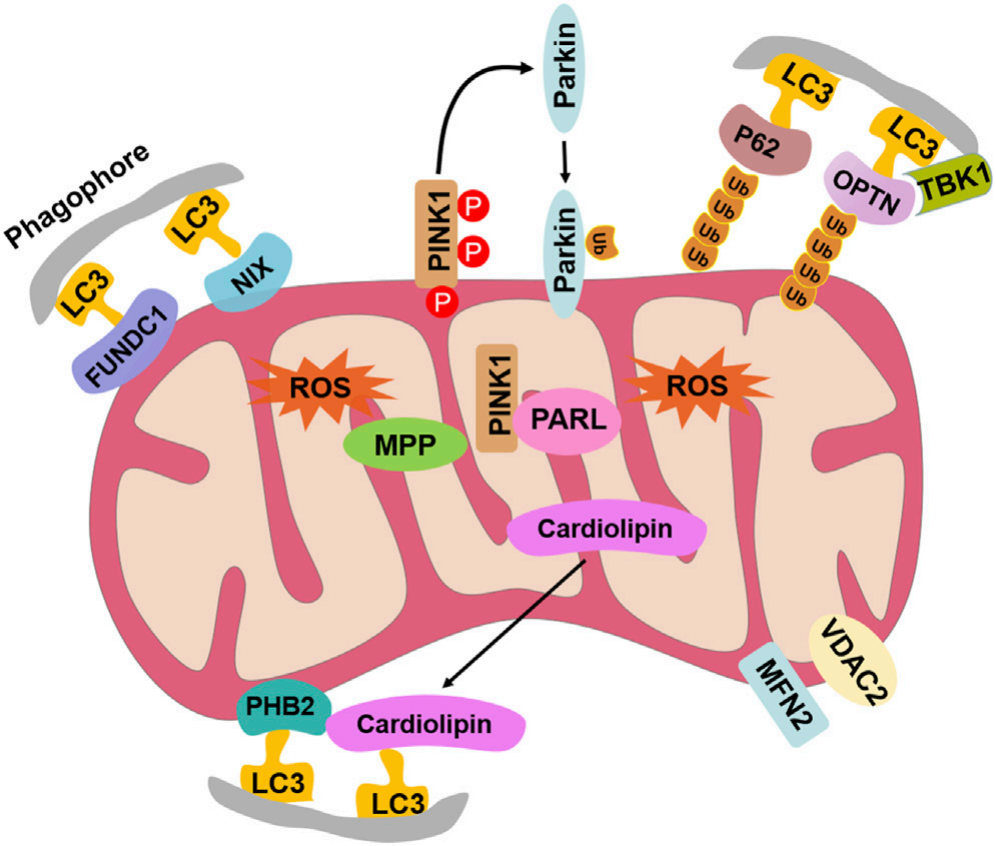
Molecular mitophagy pathway of the Pacific oyster, *C. gigas*.

Mitophagy was observed in the hemocytes of *C. gigas* induced by CCCP, and it was constitutively detected at the mRNA levels in the tested tissues of the Pacific oyster. A total of 10 genes in the mitophagy pathway were identified in *C. gigas*, and their corresponding domains were similar to their homologs in mammals and yeast (Figure 8). CCCP and *V. splendidus* were used as inducers to induce mitophagy, and this study also described an integrated approach to investigate the potential effects of mitophagy on pathogens.

## Data Availability

The original contributions presented in the study are included in the article/Supplementary Material; further inquiries can be directed to the corresponding authors.
