# Supplementary material for: LC3-Mediated Mitophagy After CCCP or Vibrio splendidus Exposure in the Pacific Oyster Crassostrea gigas
Source: Front Cell Dev Biol. 2022 May 20;10:885478. doi: 10.3389/fcell.2022.885478 (PMC9163569; doi:10.3389/fcell.2022.885478)
Supplement: Supplementary file 1 [file DataSheet1.docx]

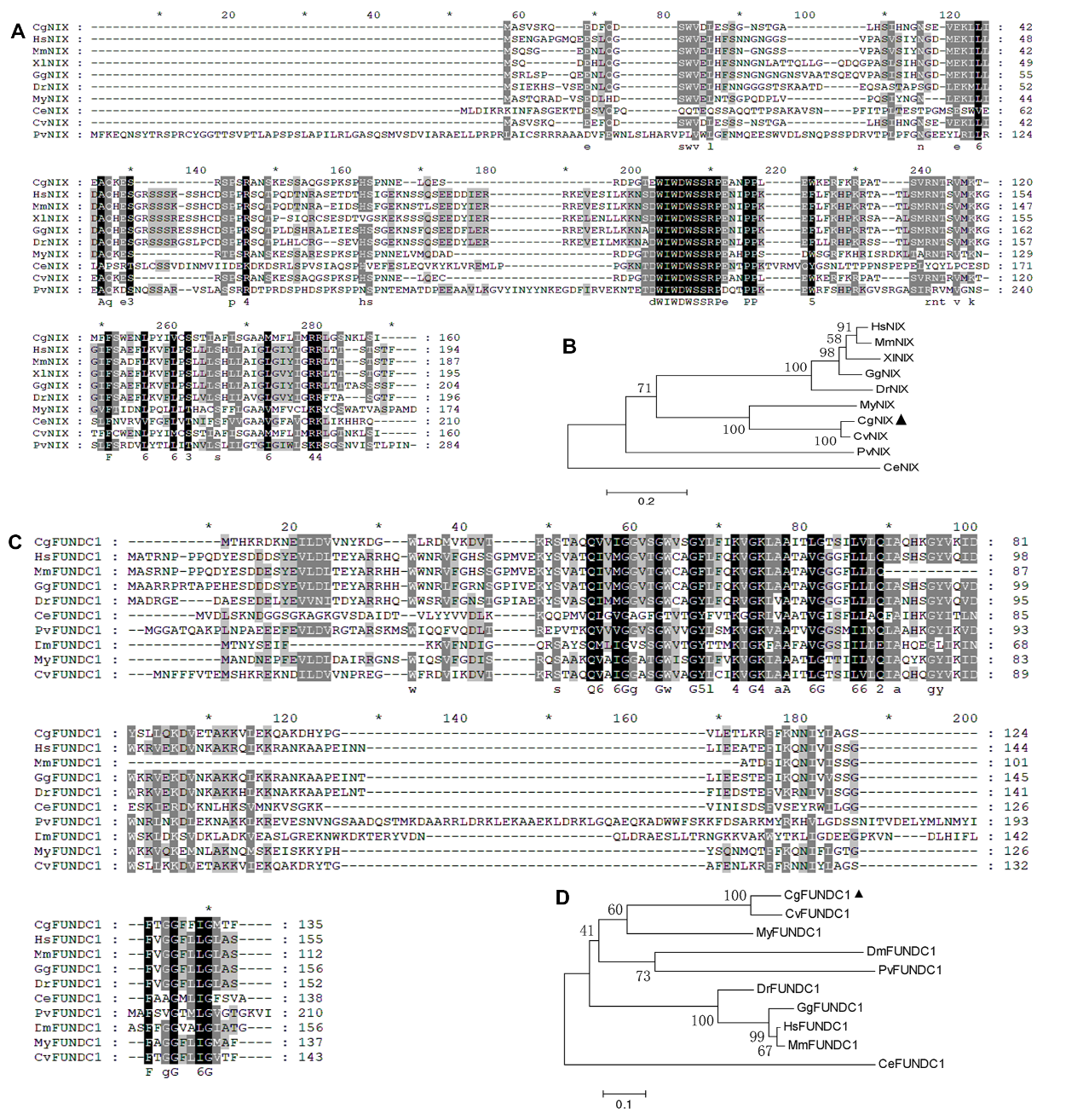


**SUPPLEMENTARY FIGURE S1 |** Alignment and phylogenic tree of *Cg*NIX and *Cg*FUNDC1 with NIXs and FUNDC1s from other species, respectively. **(A)** Black shadow indicated identical residues and gray shadow indicated similar residues in the aligned amino acids. **(B)** The numbers at the forks indicated the bootstrap. Proteins analyzed were listed below: *Cg*, *Crassostrea gigas* NIX (XP_011445562.1); *Hs*, *Homo sapiens* NIX (AAC00022.1); *Mm*, *Mus musculus* NIX (NP_033890.1); *Xl*, *Xenopus laevis* NIX (XP_018080653.1); *Gg*, *Gallus gallus* NIX (XP_421829.3); *Dr*, *Danio rerio* NIX (P_005173025.1); *Pv*, *Penaeus vannamei* NIX (ROT69101.1); *My*, *Mizuhopecten yessoensis* NIX (XP_021364080.1); *Ce*, *Caenorhabditis elegans*, NIX (AAC31574.1); *Cv*, *Crassostrea virginica* NIX (XP_022339578.1). **(C)** Black shadow indicated identical residues and gray shadow indicated similar residues in the aligned amino acids. **(D)** The numbers at the forks indicated the bootstrap. Proteins analyzed were listed below: *Cg*, *C. gigas* FUNDC1 (XP_034329090.1); *Hs*, *H. sapiens* FUNDC1 (NP_776155.1); *Mm*, *M. musculus* FUNDC1 (NP_001300674.1); *Gg*, *G. gallus* FUNDC1 (NP_001263292.1); *Dr*, *D. rerio* FUNDC1 (NP_609362.1); *Ce*, *C. elegans* FUNDC1 (NP_496404.1); *Pv*, *Penaeus vannamei* FUNDC1 (XP_027235525.1); *Dm*, *Drosophila* *melanogaster* FUNDC1 (NP_609362.1); *My*, *M. yessoensis* FUNDC1 (XP_021379239.1); *Cv*, *C. virginica* FUNDC1 (XP_022289087.1).


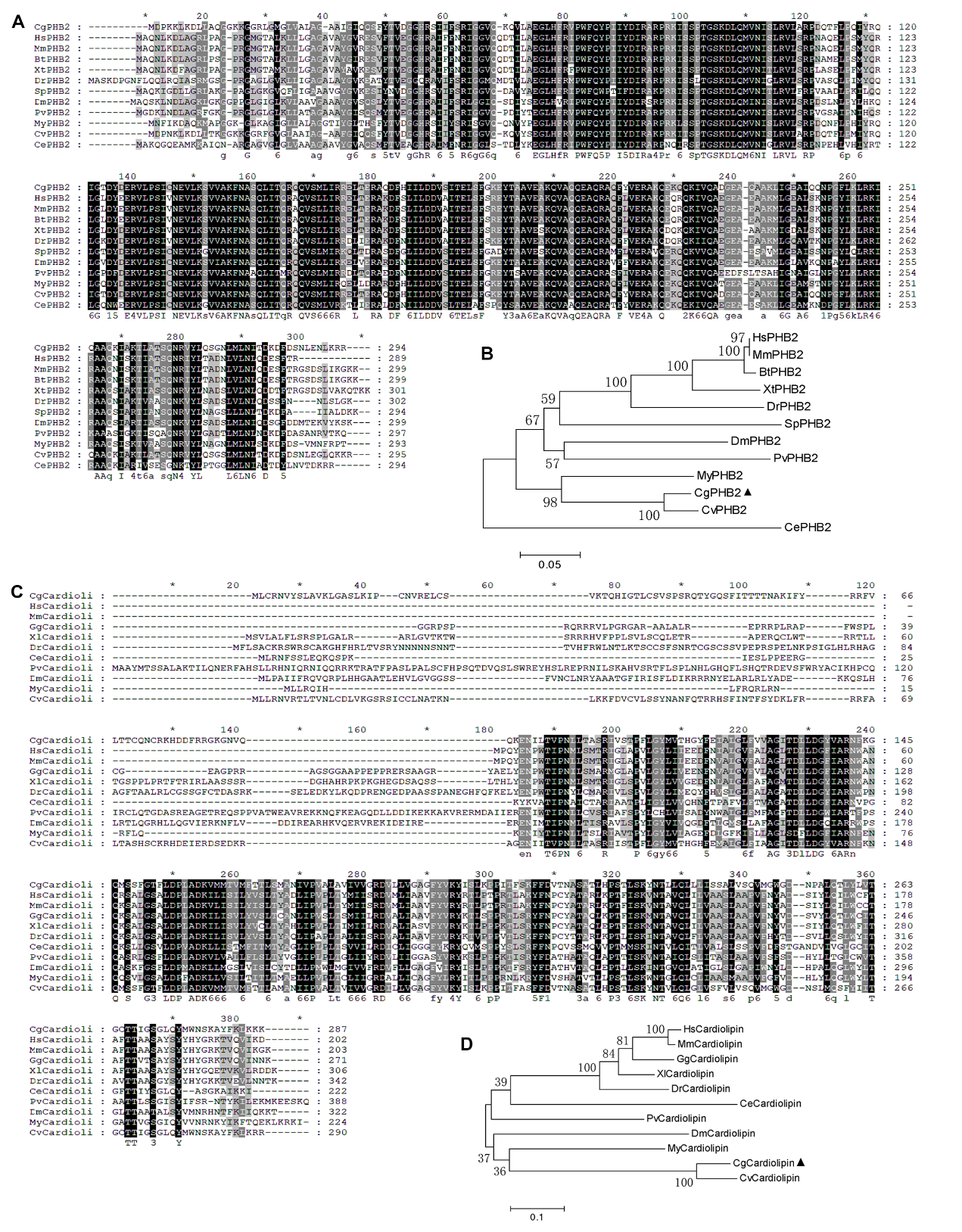


**SUPPLEMENTARY FIGURE S2 |** Alignment and phylogenic tree of *Cg*PHB2 and *Cg*Cardiolipin with PHB2s and Cardiolipins from other species, respectively. **(A)** Black shadow indicated identical residues and gray shadow indicated similar residues in the aligned amino acids. **(B)** The numbers at the forks indicated the bootstrap. Proteins analyzed were listed below: *Cg*, *C. gigas* PHB2 (XP_011427916.2); *Hs*, *H. sapiens* PHB2 (EAW88699.1); *Mm*, *M. musculus* PHB2 (NP_031557.2); *Bt*, *Bos taurus* PHB2 (NP_001039663.1); *Xt*, *Xenopus tropicalis* PHB2 (NP_001016551.1); *Dr*, *D. rerio* PHB2 (AAH59510.1); *Sp*, *Strongylocentrotus purpuratus* PHB2 (XP_030842735.1); *Dm*, *D. melanogaster* PHB2 (NP_725831.2); *Pv*, *P. vannamei* PHB2 (ROT61873.1); *My*, *M. yessoensis* PHB2 (XP_021372589.1); *Cv*, *C. virginica* PHB2 (XP_022346063.1); *Ce*, *C. elegans* PHB2 (CCD66149.1). **(C)** Black shadow indicated identical residues and gray shadow indicated similar residues in the aligned amino acids. **(D)** The numbers at the forks indicated the bootstrap. Proteins analyzed were listed below: *Cg*, *C. gigas* Cardiolipin (XP_011439682.2); *Hs*, *H. sapiens* Cardiolipin (NP_001120930.1); *Mm*, *M. musculus* Cardiolipin (XP_006500075.1); *Gg*, *G. gallus* Cardiolipin (XP_015139409.2); *Xl*, *X. laevis* Cardiolipin (NP_001090462.1); *Dr*, *D. rerio* Cardiolipin (NP_998096.1); *Ce*, *C. elegans* Cardiolipin (NP_001022547.1); *Pv*, *P. vannamei* Cardiolipin (XP_027223270.1); *Dm*, *D.* *melanogaster* Cardiolipin (NP_001262969.1); *My*, *M. yessoensis* Cardiolipin (XP_021379957.1); *Cv*, *C. virginica* Cardiolipin (XP_022333659.1).


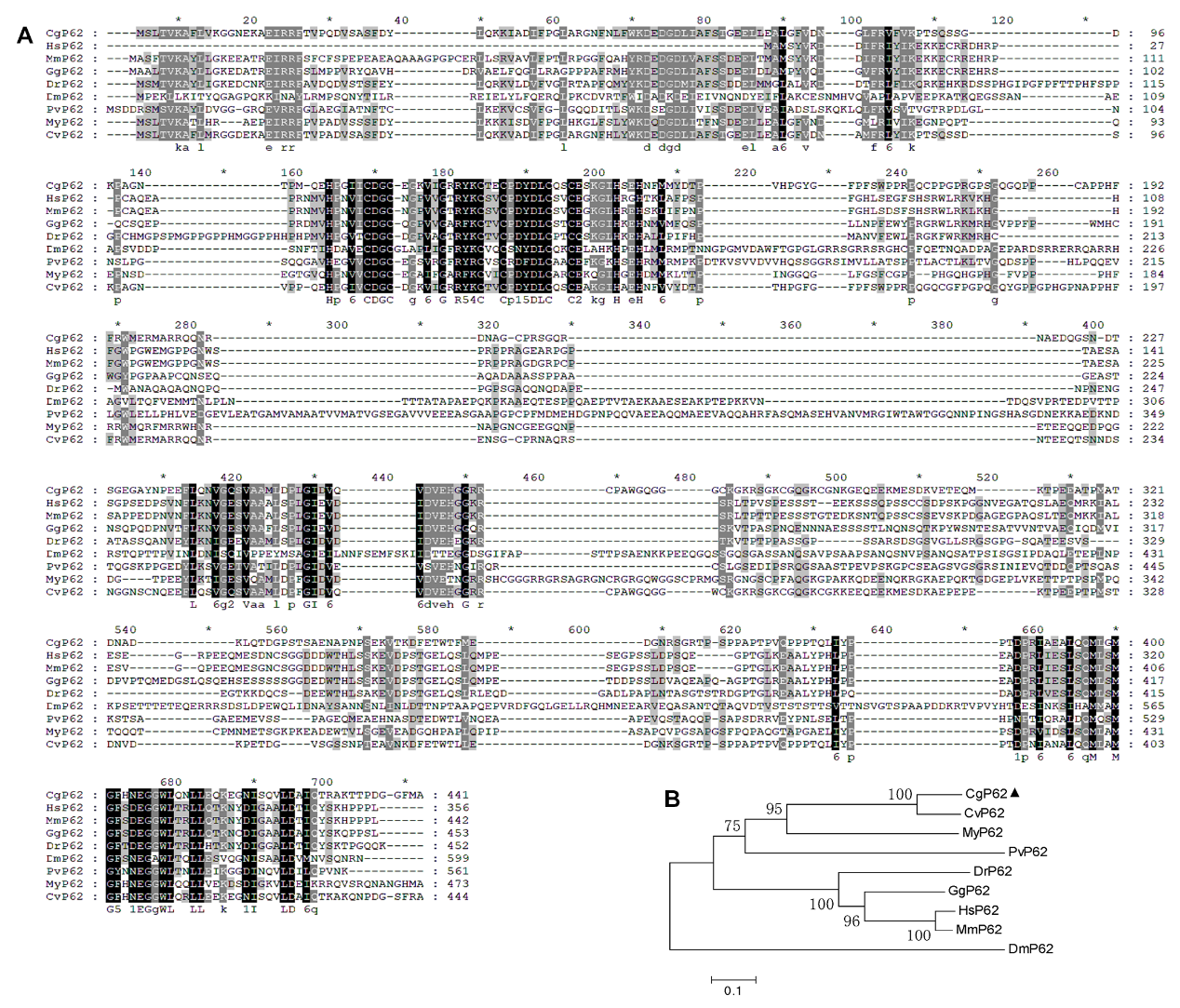


**SUPPLEMENTARY FIGURE S3 |** Alignment and phylogenic tree of *Cg*P62 with P62s from other species. **(A)** Black shadow indicated identical residues and gray shadow indicated similar residues in the aligned amino acids. **(B)** The numbers at the forks indicated the bootstrap. Proteins analyzed were listed below: *Cg*, *C. gigas* P62 (XP_011452145.1); *Hs*, *H. sapiens* P62 (AAH01874.1); *Mm*, *M. musculus* P62 (NP_035148.1); *Gg*, *G. gallus* P62 (XP_001233249.2); *Dr*, *D. rerio* P62 (NP_001299842.1); *Dm*, *D.* *melanogaster* P62 (NP_476700.1); *Pv*, *P. vannamei* P62 (ROT64427.1); *My*, *M. yessoensis* P62 (OWF55631.1); *Cv*, *C. virginica* P62 (XP_022327687.1).


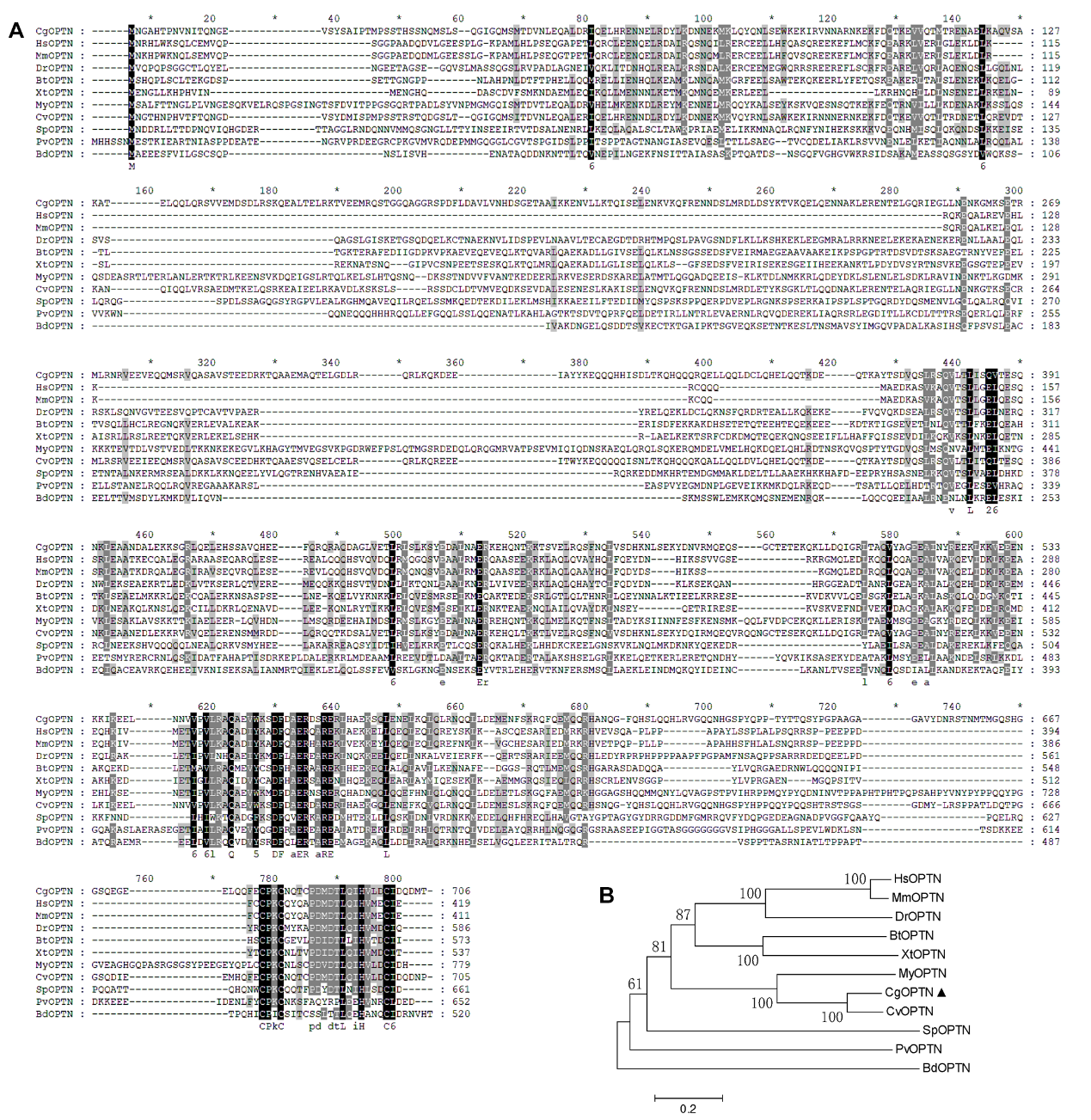


**SUPPLEMENTARY FIGURE S4 |** Alignment and phylogenic tree of *Cg*OPTN with OPTNs from other species. **(A)** Black shadow indicates identical residues and gray shadow indicates similar residues in the aligned amino acids. **(B)** The numbers at the forks indicated the bootstrap. Proteins analyzed were listed below: *Cg*, *C. gigas* OPTN (XP_034306379.1), *Hs*, *H. sapiens* OPTN (NP_001093327.1); *Mm*, *M. musculus* OPTN (NP_001154896.1); *Dr*, *D. rerio* OPTN (NP_001014366.1); *Bd*, *Bactrocera dorsalis* OPTN (JAC42003.1); *Bt*, *B. taurus* OPTN (NP_001029774.1); *Xt*, *X. tropicalis* OPTN (XP_017947826.1); *Pv*, *P. vannamei* OPTN (ROT84077.1); *My*, *M. yessoensis* OPTN (OWF48322.1); *Cv*, *C. virginica* OPTN (XP_022302308.1); *Sp*, *S. purpuratus* OPTN (XP_783059.2).


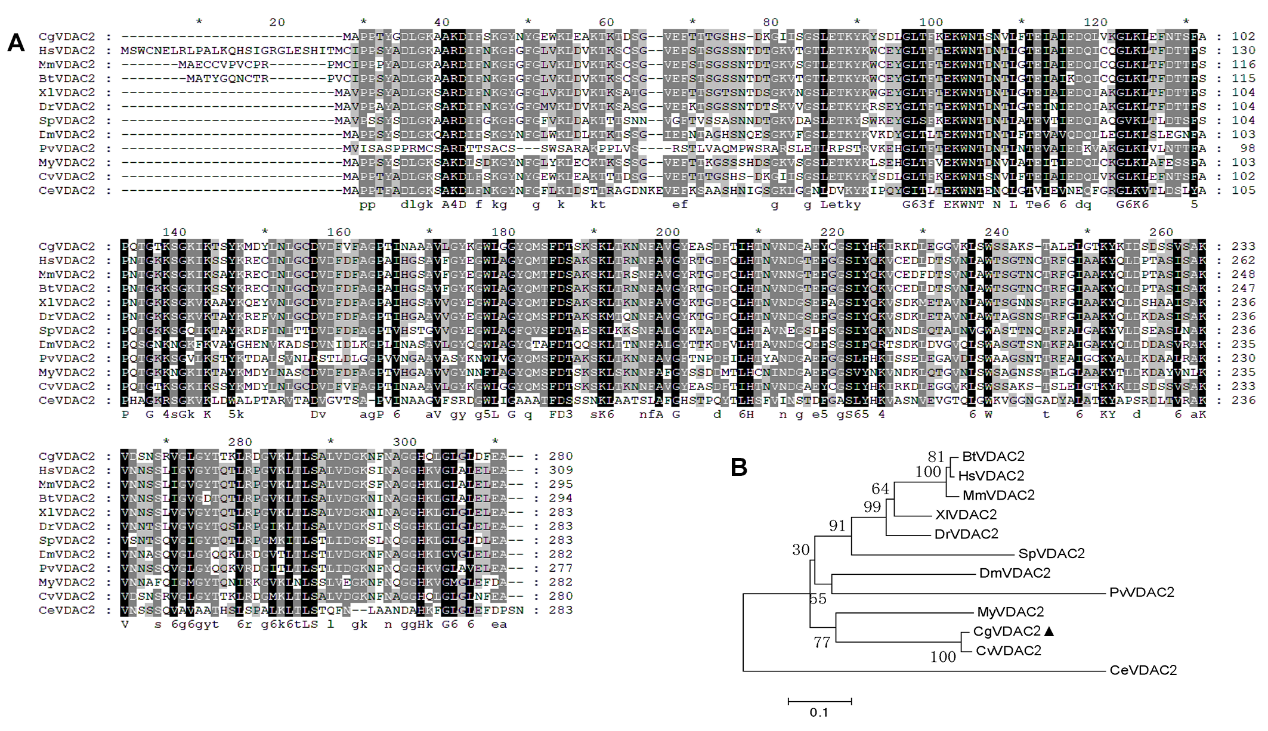


**SUPPLEMENTARY FIGURE S5 |** Alignment and phylogenic tree of *Cg*VDAC2 with VDAC2s from other species. **(A)** Black shadow indicates identical residues, and gray shadow indicates similar residues in the aligned amino acids. **(B)** The numbers at the forks indicated the bootstrap. Proteins analyzed are listed below: *Cg*, *C. gigas* VDAC2 (NP_001292225.1); *Hs*, *H. sapiens* VDAC2 (NP_001171712.1); *Mm*, *M. musculus* VDAC2 (NP_035825.1); *Bt*, *B. taurus* VDAC2 (DAA29533.1); *Dm*, *D. melanogaster* VDAC2 (NP_001033899.1); *Dr*, *D. rerio* VDAC2 (NP_955879.1); Xl, *X. laevis* (NP_001089399.1); *Pv*, *P. vannamei* VDAC2 (XP_027213642.1); *Sp*, *S. purpuratus* VDAC2 (XP_030834916.1); *My*, *M. yessoensis* VDAC2 (XP_021351930.1); *Cv*, *C. virginica* VDAC2 (XP_022316203.1); *Ce*, *C. elegans* VDAC2 (NP_501211.1).


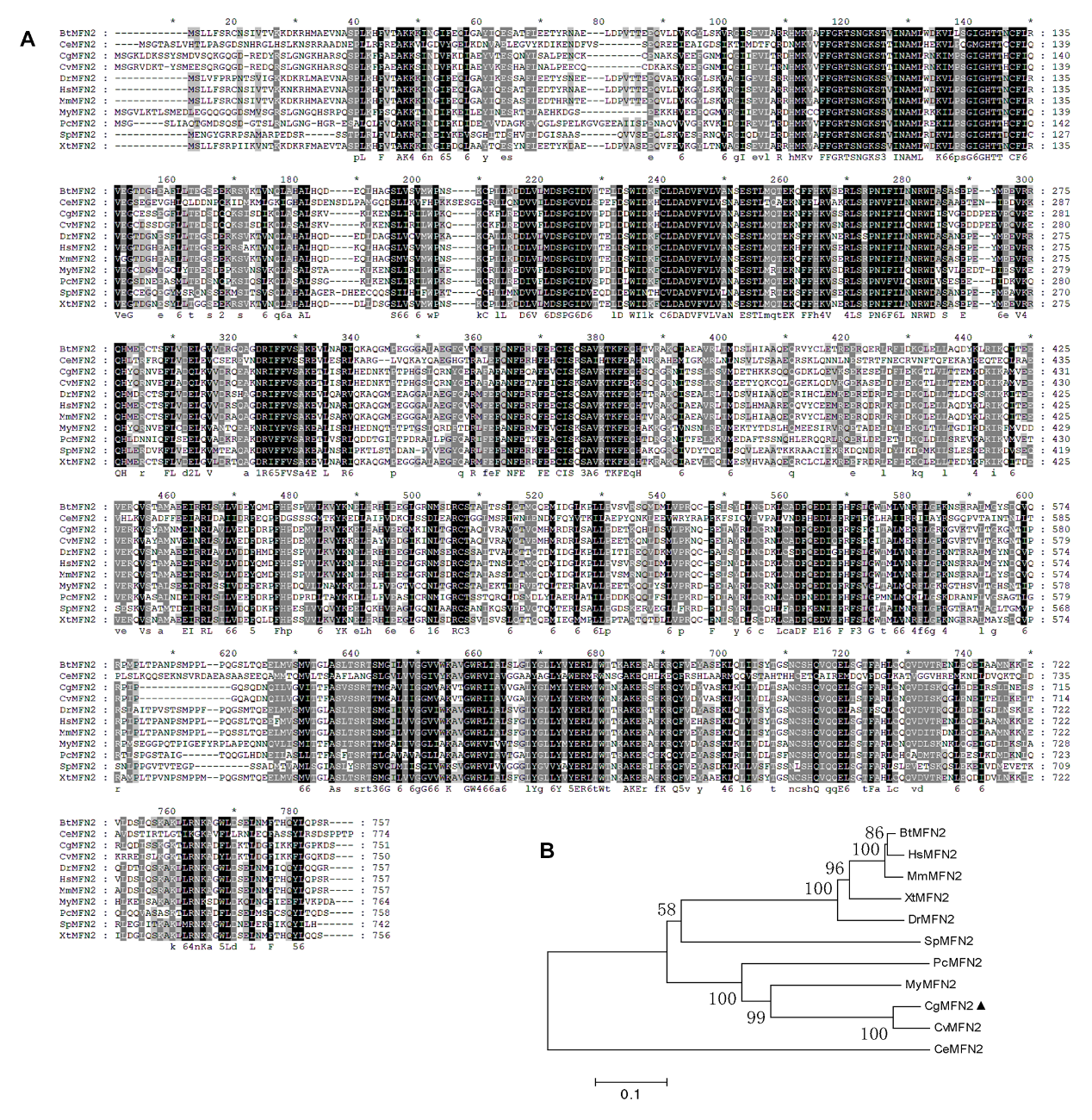


**SUPPLEMENTARY FIGURE S6 |** Alignment and phylogenic tree of *Cg*MFN2 with MFN2s from other species. **(A)** Black shadow indicated identical residues, and gray shadow indicated similar residues in the aligned amino acids. **(B)** The numbers at the forks indicated the bootstrap. Proteins analyzed were listed below: *Cg*, *C. gigas* MFN2 (XP_011449174.2); *Hs*, *H. sapiens* MFN2 (NP_001121132.1); *Mm*, *M. musculus* MFN2 (NP_001272849.1); *Bt*, *B. taurus* MFN (NP_001177198.1); *Xt*, *X. tropicalis* MFN2 (XP_031760947.1); *Dr*, *D. rerio* MFN2 (NP_001121726.1); *Sp*, *S. purpuratus* MFN2 (XP_030847518.1); *My*, *M. yessoensis* MFN2 (XP_021353688.1); *Cv*, *C. virginica* MFN2 (XP_022336819.1); *Pc*, *P. canaliculata* MFN2 (XP_025082569.1); *Ce*, *C. elegans* MFN2 (NP_495161.1).


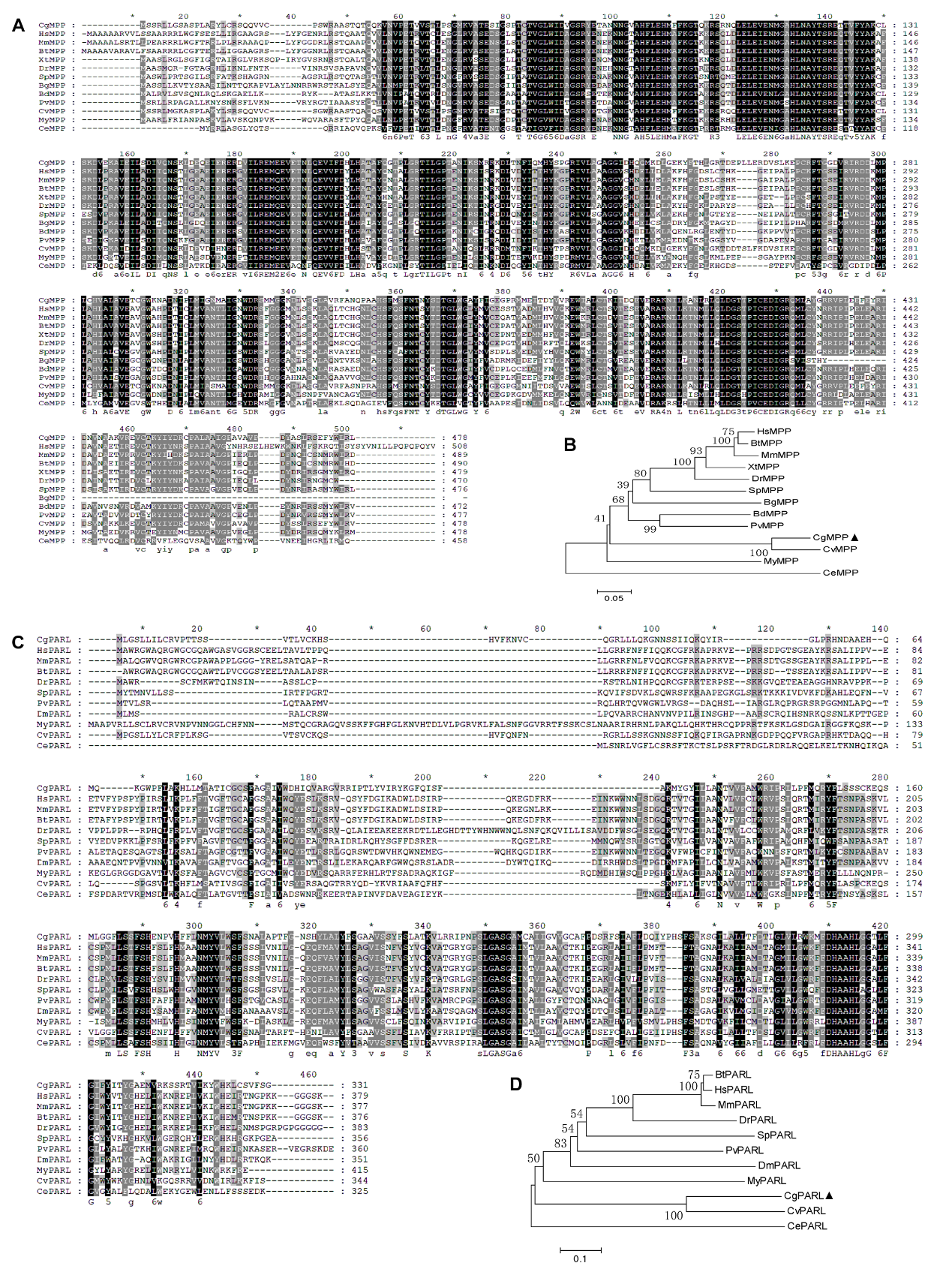


**SUPPLEMENTARY FIGURE S7 |** Alignment and phylogenic tree of *Cg*MPP and *Cg*PARL with MPPs and PARLs from other species, respectively. **(A)** Black shadow indicated identical residues, and gray shadow indicated similar residues in the aligned amino acids. **(B)** The numbers at the forks indicated the bootstrap. Proteins analyzed are listed below: *Cg*, *C. gigas* MPP (XP_034304795.1); *Hs*, *H. sapiens* MPP (XP_005250774.1); *Mm*, *M. musculus* MPP (NP_082707.1); *Bt*, *B. taurus* MPP (NP_001029785.1); *Xt*, *X. tropicalis* MPP (NP_001039103.1); *Bd*, *B. dorsalis* MPP (JAC42427.1); *Dr*, *D. rerio* MPP (NP_001012514.1); *Sp*, *S. purpuratus* MPP (XP_011678827.1); *My*, *M. yessoensis* MPP (XP_021344019.1); *Cv*, *C. virginica* MPP (XP_022302542.1); *Pv*, *P. vannamei* MPP (XP_027214917.1); *Bg*, *Biomphalaria glabrata* MPP (XP_013078886.1); *Ce*, *C. elegans* MPP (NP_501576.2). **(C)** Black shadow indicated identical residues, and gray shadow indicated similar residues in the aligned amino acids. **(D)** The numbers at the forks indicated the bootstrap. Proteins analysed were listed below: *Cg*, *C. gigas* PARL (XP_011419537.2); *Hs*, *H. sapiens* PARL (NP_061092.3); *Mm*, *M. musculus* PARL (NP_001005767.1); *Bt*, *B. taurus* PARL (ABG81447.1); *Dr*, *D. rerio* PARL (NP_001014320.1); *Dm*, *D. melanogaster* PARL (NP_001286324.1); *Pv*, *P. vannamei* PARL (XP_027229585.1); *Sp*, *S. purpuratus* PARL (XP_781024.2); *My*, *M. yessoensis* PARL (XP_021365722.1); *Cv*, *C. virginica* PARL (XP_022343884.1); *Ce*, *C. elegans* PARL (NP_491125.4).

**SUPPLEMENTARY TABLE S1 |** The sequence features of genes in the mitophagy pathway.


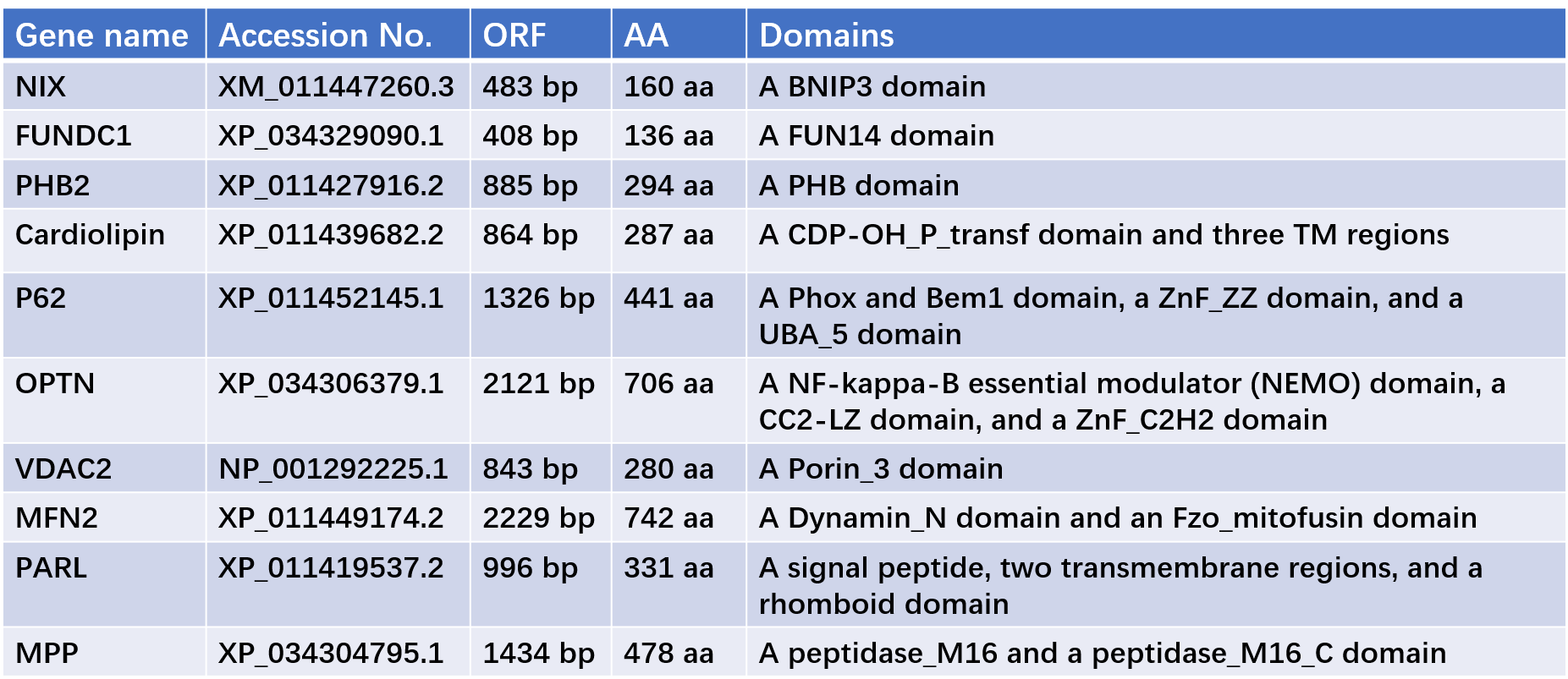


**SUPPLEMENTARY TABLE S2 |** The tissue distribution of genes in oysters.


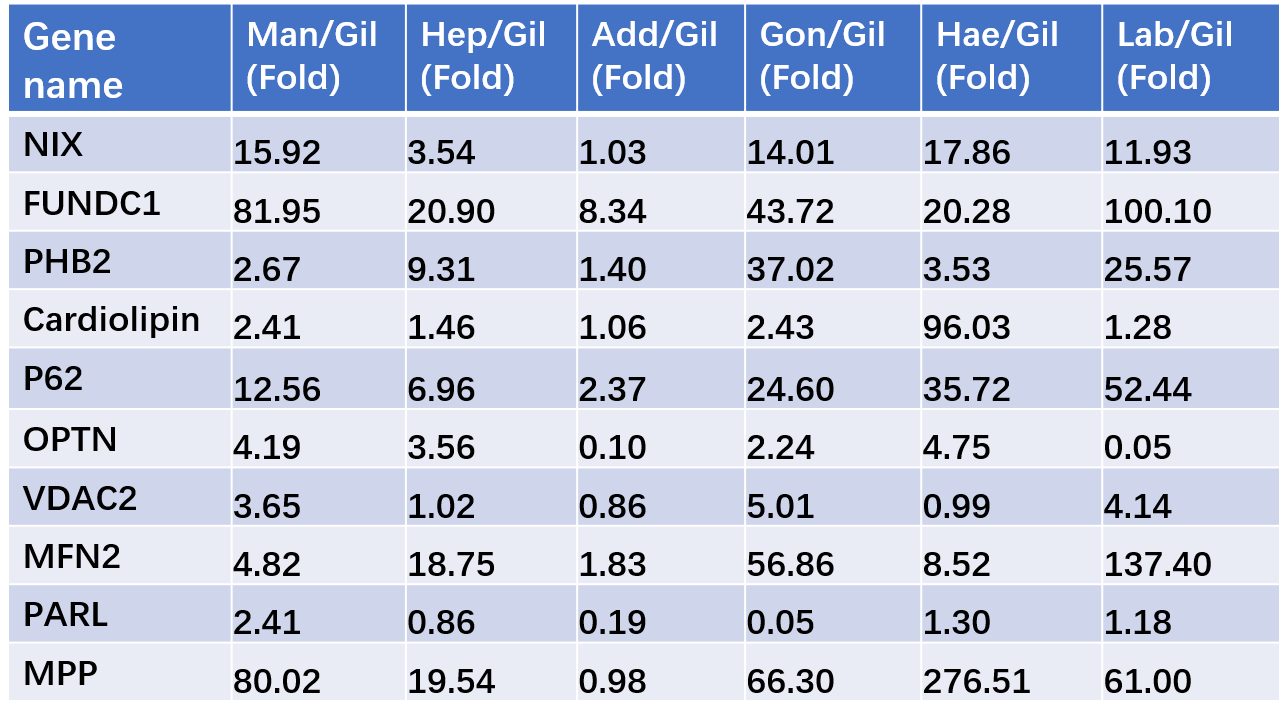


Man: mantle; Gil: gills; Hep: hepatopancreas; Add: adductor muscle; Gon: gonad; Hae: haemocytes; Lab: labial palps.

**SUPPLEMENTARY TABLE S3 |** Sequences of the primers used in this study.

| **Primer** | **Sequence (5'-3')** |
| --- | --- |
| **Clone primers** |  |
| *Cg*NIX-F | ATGGCGTCTGTGTCAAAACAG |
| *Cg*NIX-R  *Cg*FUNDC1-F  *Cg*FUNDC1-R  *Cg*PHB2-F  *Cg*PHB2-R  *Cg*P62-F  *Cg*P62-R  *Cg*VDAC2-F  *Cg*VDAC2-R  *Cg*MFN2-F  *Cg*MFN2-R  *Cg*PARL-F  *Cg*PARL-R  *Cg*MPP-F  *Cg*MPP-R  *Cg*OPTN*-*F | GATGCTCAACTTGTTACTTCC  ATGACACACAAGAGGGATAAG  TCTTCCAGTGTCAGCTGTTCA  ATGGATCCCAAGAAATTGAAA  TCGTCTCTTCAGATTCTC  ATGTCACTTACTGTAAAAGCA  GGCCATGAAGCCGCCATCAGG  ATGGCTCCCCCGACATATGGT  GGCCTCGAAGTCCAGACCGAG  ATGTCAGGAAAATTAGACAAA  ATCTTTTGGACCCAAAAA  ATGCTAGGATCTTTGTTAATT  TCCACTAAAAACACTGCA  ATGTCGTCTCGACTGTTGGGA  TGAGTCTCAGCCAGTAGAACTC  ATGAATGGAGCCCACACGCCC |
| *Cg*OPTN*-*R | GGTCATGTCCTGGTCGATACA |
| **RT-PCR primers** |  |
| *Cg*NIX-RT-F  *Cg*NIX-RT-R | GGATCTGGAATCGAGCGGAA  TTGGGCTGTGAGGACTCTTG |
| *Cg*FUNDC1-RT-F  *Cg*FUNDC1-RT-R  *Cg*PHB2-RT-F  *Cg*PHB2-RT-R  *Cg*Cardiolipin-RT-F  *Cg*Cardiolipin-RT-R  *Cg*OPTN-RT-F  *Cg*OPTN-RT-R  *Cg*VDAC2-RT-F | TCATCGGCGGAGTATCAGGA  GCTGTGCAATCTGAAGAACCA  CCTCCCAACTTATTACAC  TCAACTTCAAAAATCCAG  TTTGGCTGTTAAACTCGGCG  CGTATGTCTGACGACTGGGG  GGCTGTCCCTGAAGAGTT  CTGCTTCTCTGTCTCCGT  GCTCGTGAAAGGATTGAAACT |
| *Cg*VDAC2-RT-R | TTGGTGTGAATGGTAAAG |
| *Cg*MFN2-RT-F | ACCAGTTTGAGATTGCGT |
| *Cg*MFN2-RT-R | ATGAGTCCGTAGAGGGCT |
| *Cg*MPP-RT-F | TGCGTCCTTACAGCACACAAC |
| *Cg*MPP-RT-R | CCTACATCAATCCACAAACCC |
| *Cg*PARL-RT-F | AGGGTTTCTCTCCTCTTTCAG |
| *Cg*PARL-RT-R | GCAACGCAAGGATACCTGACT |
| *Cg*P62-RT-F | AAGCTGCAGACAGATGGACC |
| *Cg*P62-RT-R | CTTGGCTCTGGTCTGGATGG |
| *Cg*EF-RT-F | AGTCACCAAGGCTGCACAGAAAG |
| *Cg*EF-RT-R | TCCGACGTA TTTCTTTGCGATGT |
